# Optical Force and Torque on a Graphene-Coated Gold Nanosphere by a Vector Bessel Beam

**DOI:** 10.3390/mi13030456

**Published:** 2022-03-17

**Authors:** Bing Yan, Xiulan Ling, Renxian Li, Jianyong Zhang, Chenhua Liu

**Affiliations:** 1 Shool of Information and Communication Engineering, North University of China, Taiyuan 030051, China; nmlxlmiao@126.com; 2 School of Physics and Optoelectronic Engineering, Xidian University, Xi’an 710071, China; rxli@mail.xidian.edu.cn; 3 School of Computing, Engineering and Digital Technologies, Teesside University, Middlesbrough TS1 3BA, UK; j.zhang@tees.ac.uk; 4 Application Science Institute, Taiyuan University of Science and Technology, Taiyuan 030024, China; lchygs78@163.com

**Keywords:** optical force, optical torque, vector Bessel beam, graphene-coated gold nanosphere, generalized Lorenz–Mie theory, polarization

## Abstract

In the framework of the generalized Lorenz–Mie theory (GLMT), the optical force and torque on a graphene-coated gold nanosphere by a vector Bessel beam are investigated. The core of the particle is gold, whose dielectric function is given by the Drude–Sommerfeld model, and the coating is multilayer graphene with layer number *N*, whose dielectric function is described by the Lorentz–Drude model. The axial optical force $F_{z}$ and torque $T_{z}$ are numerically analyzed, and the effects of the layer number *N*, wavelength λ, and beam parameters (half-cone angle $\alpha_{0}$, polarization, and order *l*) are mainly discussed. Numerical results show that the optical force and torque peaks can be adjusted by increasing the thickness of the graphene coating, and can not be adjusted by changing $\alpha_{0}$ and *l*. However, $\alpha_{0}$ and *l* can change the magnitude of the optical force and torque. The numerical results have potential applications involving the trapped graphene-coated gold nanosphere.

## 1. Introduction

Light carries both linear and orbit angular momentum. During the interaction between light and small particles, the angular momentum will be transferred from light to particle, and the particles will experience optical force and torque. Optical tweezers, which are based on the optical force and torque, have been used for the manipulation and rotation of microscopic objects, and have found many particularly appealing applications in the field of biomedical engineering. Traditional optical tweezers use Gaussian beams, which suffer from diffraction. To overcome the diffraction, some novel manipulation techniques based on nondiffracting beams [1,2,3,4] have been developed. Bessel beams [5,6,7,8], a typical nondiffracting beam, can simultaneously trap and manipulate many particles in multiple planes because of their unique properties of nondiffraction and self-healing. In addition, by adjusting the beam parameters including half-cone angle, beam order, and polarization, Bessel beams can exert pulling force [9,10,11,12,13,14,15,16,17,18,19,20,21,22,23,24,25,26,27,28,29,30,31,32,33,34,35,36] and negative optical torque [3,37,38,39,40,41] on particles.

In recently years, gold nanoparticles have proven to be an excellent tool for optical-tweezer-based micromanipulation due to their properties of large polarizability, relatively low cytotoxicity, and localized surface plasmon resonance (LSPR) [42,43,44], and optically trapped gold nanoparticles have wide applications in bioengineering such as nanosensors [45,46], bioimaging [47,48,49,50], diagnostics [51,52,53], etc. For instance, gold nanoaperture optical tweezers have been used for the manipulation, sensing, and spectroscopy of biological nanoparticles below 50 nm in size [54]. Furthermore, the gold nanoparticles have been used to enhance bio-optical imaging, and to improve its resolution, sensitivity, and penetration depth [55]. Because of the LSPR in the visible or near-infrared region, nanoparticles can play crucial roles in optical tweezers by enhancing the gradient force if the trapping laser is tuned to the long-wavelength side [56].

In recent years, graphene has attracted much attention because of its extraordinary electro-optical properties. Adding a graphene coating on gold nanoparticle shows many advantages and has many novel applications. The graphene can protect the nanoparticles since it can avoid the oxidation of nanoparticles [44]. A graphene-coated gold nanoparticle shows high thermal stability and unique optical properties, and has potential applications for photothermal therapy (PTT) [57]. Taking graphene-coated spherical nanoparticles as the unit cells, a tunable optical metasurface was realized [58]. In these applications, the coating is monolayer graphene, which is considered a thin sheet with a complex surface conductivity. Research shows that using multilayer graphene coating has many novel applications. The increasing of graphene coating thickness (or layer number of graphene coating) significantly shift the resonance wavelength, and the spectral properties of the system enhances the applicability for sensing applications [44]. By increasing the graphene coating thickness, the extinction peak of graphene-coated gold nanoparticle can be adjusted. A gold nanoparticle coated with multilayer graphene has photothermal applications, since graphene with a controllable thickness has excellent robustness and stability in a biological environment [57].

Many studies have been devoted to the optical force on a graphene-coated particle, with emphasis on the effect of the graphene coating. Caused by the local field enhancement due to surface plasmon resonance and the nonlinear response of graphene, a tunable optical force on graphene-coated nanoparticles can be observed [59]. With a graphene coating, a tunable optical force on a microparticle can be realized under the illumination of Gaussian [60] or Bessel beams [61]. However, in these studies, the core of the particles was dielectric. In this paper, the optical force and torque on a graphene-coated gold nanosphere by a vector Bessel beam are investigated.

The rest of the paper is organized as follows. A general theory of the optical force and torque on a graphene-coated gold nanosphere by a vector Bessel beam is given in Section 2. The core is a gold sphere, whose dielectric function is given by the Drude–Sommerfeld model. The coating is multilayer graphene with layer number *N*, whose dielectric function is described by the Lorentz–Drude model. The generalized Lorenz–Mie theory for optical force and torque are given. The scattering coefficients are the traditional Mie scattering coefficients for a coated sphere, and the beam-shaped coefficients are given based on the angular spectrum decomposition method and multipole expansion. The optical force and torque are expressed in terms of the surface integration of a Maxwell stress tensor. Section 3 discusses some numerical results of the optical force and torque exerted on a graphene-coated gold nanosphere, with emphasis on the effects of the graphene coating thickness and the beam parameters including half-cone angle $\alpha_{0}$, order *l*, and polarization. A conclusion of the present work is outlined in Section 4.

## 2. Theory

**Figure 1:**
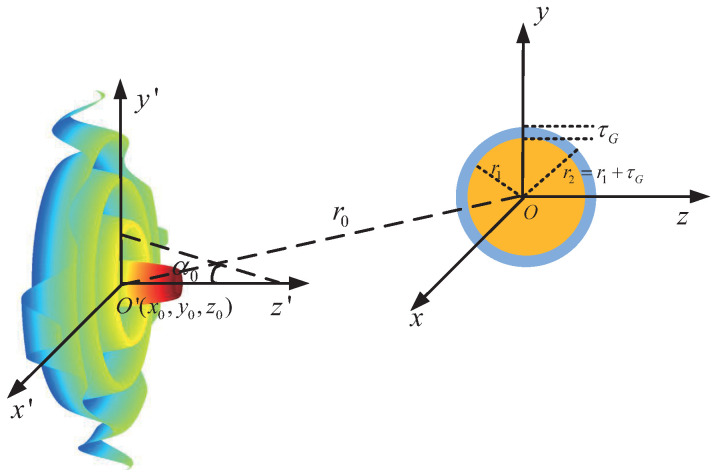
Geometry of a graphene-coated gold nanosphere illuminated by a vector Bessel beam.

Consider a graphene-coated gold nanosphere illuminated by a vector Bessel beam, as shown in Figure 1. The center of the particle is located at *O*, which is the origin of the coordinate system O−xyz. The center of the beam is located at $O^{\prime }$, which is the origin of the coordinate system $O^{\prime }-x^{\prime }y^{\prime }z^{\prime }$. The coordinates of the beam center $O^{\prime }$ in O−xyz are $\left(x_{0},y_{0},z_{0}\right)$. The refractive index of the surrounding media is $m_{3}$, which is assumed to be 1 in our calculation.

The core of the particle is a gold nanosphere, whose radius is $r_{1}$ and whose dielectric function is given by the Drude–Sommerfeld model [57,62]:

(1) $$\varepsilon_{1}\left(\omega \right)=\varepsilon_{b}-\frac{\omega_{Au}^{2}}{\omega \left(\omega +i\gamma_{Au}\right)}$$

with

(2) $$\gamma_{Au}=\gamma_{bulk}+A\frac{v_{F}}{a_{eff}}$$

where ω is the angular frequency, $\varepsilon_{b}$ is the phenomenological parameter, $\omega_{Au}$ is the bulk plasmon frequency of gold, $\gamma_{bulk}$ is the frequency of electron collisions, *A* is a constant parameter for matching theoretical and experimental results, $v_{F}$ is the Fermi velocity of the electron, and $a_{eff}$ is the effective radius of the particle. In our calculation, they are $\varepsilon_{b}=9.8$, $\omega_{Au}=9$ eV, $\gamma_{bulk}=0.066$ eV, A=0.25, and $v_{F}=1.4\times 10^{6}$ m/s.

The coating is multilayer graphene with layer number *N*, and its thickness is $\tau_{G}=N\tau_{sg}$. $\tau_{sg}$ is the thickness of monolayer graphene, and is taken to be 0.335 nm in our calculation. The dielectric function of graphene can be given by the Lorentz–Drude mode [57,63]:

(3) $$\varepsilon \left(\omega \right)=\varepsilon_{\infty }-\frac{\omega_{p}{}^{2}}{\omega^{2}+i\omega \gamma }+\sum_{j=1}^{N}\frac{\Delta \varepsilon_{j}\Omega_{j}^{2}}{\Omega_{j}^{2}-\omega^{2}-i\omega \Gamma_{j}}$$

In our calculation, the parameters in Equation (3) were: $\varepsilon_{\infty }=1.964$, $\Delta \varepsilon_{j}=\left(6.99,1.69,1.53\right)$, $\hbar \omega_{p}=6.02$ eV, ℏγ=4.52 eV, $\hbar \Omega_{j}=\left(3.14,4.03,4.59\right)$ eV, and $\hbar \Gamma_{j}=\left(7.99,2.01,0.88\right)$ eV.

The incident beam is an *l*-order vector Bessel beam propagating along the *z* axis, and its electric field can be given by the angular spectrum decomposition method (ASDM):

(4) $$E\left(r,\theta ,\phi \right)=\int_{\beta =0}^{2\pi }E_{pw0}Q^{u}e^{il\beta }e^{ik\;\cdot \;(r-r_{0})}d\beta$$

where k is the wave vector, r is the position vector, and $r_{0}=\left(x_{0},y_{0},z_{0}\right)$ is the center of the beam. The vector complex polarization function $Q^{u}$ with the superscript *u* denoting the polarization state is [64,65]

(5) $$Q^{u}=\left[\begin{array}{c} \begin{array}{c} p_{x}\left(\cos\alpha_{0}\cos^{2}\beta +\sin^{2}\beta \right)-p_{y}\left(1-\cos\alpha_{0}\right)\sin\beta \cos\beta \end{array}\\ \begin{array}{c} -p_{x}\left(1-\cos\alpha_{0}\right)\sin\beta \cos\beta +p_{y}\left(\cos\alpha_{0}\sin^{2}\beta +\cos^{2}\beta \right) \end{array}\\ \begin{array}{c} -p_{x}\sin\alpha_{0}\cos\beta -p_{y}\sin\alpha_{0}\sin\beta \end{array} \end{array}\right]$$

where $\alpha_{0}$ is the half-cone angle of the Bessel beam. By choosing $p_{x}$ and $p_{y}$, we can obtain the electric field of the vector Bessel beam with various polarizations using Equation (4). For convenience, the electric fields of Bessel beams with linear, circular, radial, azimuthal, and mixed polarizations are given in Appendix A.

According to the generalized Lorenz–Mie theory (GLMT), the incident and scattered electric fields can be expanded using VSWFs and BSCs ($g_{n,TE}^{m}$, $g_{n,TM}^{m}$) [66,67]:

(6) $$E_{i}=\sum_{n=1}^{\infty }\sum_{m=-n}^{n}c_{n}^{pw}\left[g_{n,TM}^{m,u}N_{mn}^{\left(1\right)}\left(kr\right)+ig_{n,TE}^{m,u}M_{mn}^{\left(1\right)}\left(kr\right)\right]$$

(7) $$H_{i}=-\frac{ik}{\omega }\sum_{n=1}^{\infty }\sum_{m=-n}^{n}c_{n}^{pw}\left[g_{n,TM}^{m,u}M_{mn}^{\left(1\right)}\left(kr\right)+ig_{n,TE}^{m,u}N_{mn}^{\left(1\right)}\left(kr\right)\right]$$

(8) $$E_{s}=\sum_{n=1}^{\infty }\sum_{m=-n}^{n}c_{n}^{pw}\left[A_{n}^{m,u}N_{mn}^{\left(4\right)}\left(kr\right)+iB_{n}^{m,u}M_{mn}^{\left(4\right)}\left(kr\right)\right]$$

(9) $$H_{s}=-\frac{ik}{\omega }\sum_{n=1}^{\infty }\sum_{m=-n}^{n}c_{n}^{pw}\left[A_{n}^{m,u}M_{mn}^{\left(4\right)}\left(kr\right)+iB_{n}^{m,u}N_{mn}^{\left(4\right)}\left(kr\right)\right]$$

where k=2π/λ=ω/c is the wavenumbers. $c_{n}^{pw}$ is defined by

(10) $$c_{n}^{pw}=i^{n+1}\frac{2n+1}{n(n+1)}.$$

According to the GLMT, we can obtain the relation between the expansion coefficients of scattered fields $\left(A_{n}^{m,u},B_{n}^{m,u}\right)$ and BSCs $\left(g_{n,TM}^{m,u},g_{n,TE}^{m,u}\right)$, which have been derived in our previous papers and are given in Appendix B for convenience:

(11) $$A_{n}^{m,u}=a_{n}g_{n,TM}^{m,u},\;B_{n}^{m,u}=b_{n}g_{n,TE}^{m,u}$$

where $a_{n}$ and $b_{n}$ are traditional Mie scattering coefficients for a coated sphere:

(12) $$\;a_{n}\;=\;\frac{\psi_{n}\left(y\right)\left[\psi_{n}^{\prime }\left(m_{2}y\right)-A_{n}\chi_{n}^{\prime }\left(m_{2}y\right)\right]-m_{2}\psi_{n}^{\prime }\left(y\right)\left[\psi_{n}\left(m_{2}y\right)-A_{n}\chi_{n}\left(m_{2}y\right)\right]}{\xi_{n}\left(y\right)\left[\psi_{n}^{\prime }\left(m_{2}y\right)-A_{n}\chi_{n}{}^{\prime }\left(m_{2}y\right)\right]-m_{2}\xi_{n}{}^{\prime }\left(y\right)\left[\psi_{n}\left(m_{2}y\right)-A_{n}\chi_{n}\left(m_{2}y\right)\right]}$$

(13) $$\;b_{n}\;=\;\frac{m_{2}\psi_{n}\left(y\right)\left[\psi_{n}{}^{\prime }\left(m_{2}y\right)-B_{n}\chi_{n}{}^{\prime }\left(m_{2}y\right)\right]-\psi_{n}{}^{\prime }\left(y\right)\left[\psi_{n}\left(m_{2}y\right)-B_{n}\chi_{n}\left(m_{2}y\right)\right]}{m_{2}\xi_{n}\left(y\right)\left[\psi_{n}{}^{\prime }\left(m_{2}y\right)-B_{n}\chi_{n}{}^{\prime }\left(m_{2}y\right)\right]-\xi_{n}{}^{\prime }\left(y\right)\left[\psi_{n}\left(m_{2}y\right)-B_{n}\chi_{n}\left(m_{2}y\right)\right]}$$

(14) $$\begin{array}{ccc} A_{n} & = & \frac{m_{2}\psi_{n}\left(m_{2}x\right)\psi_{n}{}^{\prime }\left(m_{1}x\right)-m_{1}\psi_{n}{}^{\prime }\left(m_{2}x\right)\psi_{n}\left(m_{1}x\right)}{m_{2}\chi_{n}\left({\tilde{n}}_{2}x\right)\psi_{n}{}^{\prime }\left(m_{1}x\right)-m_{1}\chi_{n}{}^{\prime }\left(m_{2}x\right)\psi_{n}\left(m_{1}x\right)} \end{array}$$

(15) $$\begin{array}{ccc} B_{n} & = & \frac{m_{2}\psi_{n}\left(m_{1}x\right)\psi_{n}{}^{\prime }\left(m_{2}x\right)-m_{1}\psi_{n}{}^{\prime }\left(m_{1}x\right)\psi_{n}\left(m_{2}x\right)}{m_{2}\chi_{n}{}^{\prime }\left(m_{2}x\right)\psi_{n}\left(m_{1}x\right)-m_{1}\chi_{n}\left(m_{2}x\right)\psi_{n}{}^{\prime }\left(m_{1}x\right)} \end{array}$$

where $\psi_{n}\left(x\right)=xj_{n}\left(x\right)$, $\chi_{n}\left(x\right)=-xy_{n}\left(x\right)$, $\xi_{n}\left(x\right)=xh_{n}^{\left(1\right)}\left(x\right)$ are Ricatti–Bessel functions. $x=kr_{1}$, and $y=kr_{2}$ are the dimensionless size parameters of the core and coating, respectively.

The optical force exerted on a particle by a beam is proportional to the net momentum removed from the incident beam, and can be expressed in terms of the surface integration of a Maxwell stress tensor

(16) $$<F>=<\oint_{S}\hat{n}\;\cdot \;\overset{↔}{A}dS>$$

where <> represents a time average, $\hat{n}$ the outward normal unit vector, and *S* a surface enclosing the particle. The Maxwell stress tensor $\overset{↔}{A}$ is given by

(17) $$\overset{↔}{A}=\frac{1}{4\pi }\left(\varepsilon EE+HH-\frac{1}{2}\left(\varepsilon E^{2}+H^{2}\right)\overset{↔}{I}\right)$$

where the electromagnetic fields E and H are the total fields, namely, the sum of the incident and scattered fields. Substituting the incident and scattered fields for a vector Bessel beam into Equations (16) and (17), we can get the optical force according to the GLMT [66]:

(18) $$F^{u}\left(r\right)=\frac{2m_{3}I_{0}}{c}\left[e_{x}C_{pr,x}^{u}\left(r\right)+e_{y}C_{pr,y}^{u}\left(r\right)+e_{z}C_{pr,z}^{u}\left(r\right)\right]$$

where the longitudinal ($C_{pr,z}^{u}$) and transverse ($C_{pr,x}^{u}$ and $C_{pr,y}^{u}$) radiation pressure cross sections are

(19) $$\begin{array}{cc} C_{pr,z}^{u} & =\frac{\lambda^{2}}{\pi }\sum_{n=1}^{\infty }Re\left\{\frac{1}{n+1}(A_{n}g_{n,TM}^{0,u}g_{n+1,TM}^{0,u*}\right.\\ \; & +B_{n}g_{n,TE}^{0,u}g_{n+1,TE}^{0,u*})+\sum_{m=1}^{n}\left[\frac{1}{{\left(n+1\right)}^{2}}\frac{(n+m+1)!}{(n-m)!}\right.\\ \; & \times (A_{n}g_{n,TM}^{m,u}g_{n+1,TM}^{m,u*}+A_{n}g_{n,TM}^{-m,u}g_{n+1,TM}^{-m,u*}\\ \; & +B_{n}g_{n,TE}^{m,u}g_{n+1,TE}^{m,u*}+B_{n}g_{n,TE}^{-m,u}g_{n+1,TE}^{-m,u*})\\ \; & +m\frac{2n+1}{n^{2}{\left(n+1\right)}^{2}}\frac{(n+m)!}{(n-m)!}C_{n}(g_{n,TM}^{m,u}g_{n,TE}^{m,u*}\left.\left.-g_{n,TM}^{-m,u}g_{n,TE}^{-m,u*})\right]\right\} \end{array}$$

(20) $$C_{pr,x}^{u}=Re\left(C^{u}\right)\;C_{pr,y}^{u}=Im\left(C^{u}\right)$$

with

(21) $$\begin{array}{cc} C^{u} & =\frac{\lambda^{2}}{2\pi }\sum_{n=1}^{\infty }\left\{-\frac{(2n+2)!}{{\left(n+1\right)}^{2}}F_{n}^{n+1,u}+\sum_{m=1}^{n}\frac{(n+m)!}{(n-m)!}\frac{1}{{\left(n+1\right)}^{2}}\right.\\ \; & \left[F_{n}^{m+1,u}-\frac{n+m+1}{n-m+1}F_{n}^{m,u}+\frac{2n+1}{n^{2}}(C_{n}g_{n,TM}^{m-1,u}g_{n,TE}^{m,u*}\right.\\ \; & -C_{n}g_{n,TM}^{-m,u}g_{n+1,TE}^{-m+1,u*}+C_{n}^{*}g_{n,TE}^{m-1,u}g_{n,TM}^{m,u*}\left.\left.-C_{n}^{*}g_{n,TE}^{-m,u}g_{n,TM}^{-m+1,u*})\right]\right\} \end{array}$$

and

(22) $$\begin{array}{cc} F_{n}^{m,u} & =A_{n}g_{n,TM}^{m-1,u}g_{n+1,TM}^{m,u*}+B_{n}g_{n,TE}^{m-1,u}g_{n+1,TE}^{m,u*}\\ & +A_{n}^{m*}g_{n+1,TM}^{-m,u}g_{n,TM}^{-m+1,u*}+B_{n}^{m*}g_{n+1,TE}^{-m,u}g_{n,TE}^{-m+1,u*} \end{array}$$

(23) $$\begin{array}{ccc} A_{n} & = & a_{n}+a_{n+1}^{*}-2a_{n}a_{n+1}^{*}\\ B_{n} & = & b_{n}+b_{n+1}^{*}-2b_{n}b_{n+1}^{*}\\ C_{n} & = & -i(a_{n}+b_{n+1}^{*}-2a_{n}b_{n+1}^{*}) \end{array}$$

Note that Equations (19)–(23) hold for both homogeneous and coated spheres, depending on the use of Mie scattering coefficients.

The optical torque exerted can also be expressed in terms of the Maxwell stress tensor according to the GLMT as:

(24) $$<T>=-\oint_{S}\hat{n}\;\cdot \;<\overset{↔}{A}>\times rdS$$

Substituting the electromagnetic fields into Equation (24) and after some algebra, we have

(25) $$\begin{array}{ccc} lT_{x}^{u} & = & \frac{4m_{3}}{c}\frac{\pi }{k^{3}}\sum_{n=1}^{\infty }\sum_{m=1}^{n}C_{n}^{m}R\left(A_{n}^{m,u}\right) \end{array}$$

(26) $$\begin{array}{ccc} T_{y}^{u} & = & \frac{4m_{3}}{c}\frac{\pi }{k^{3}}\sum_{n=1}^{\infty }\sum_{m=1}^{n}C_{n}^{m}I\left(A_{n}^{m,u}\right) \end{array}$$

(27) $$\begin{array}{ccc} T_{z}^{u} & = & -\frac{4m_{3}}{c}\frac{\pi }{k^{3}}\sum_{n=1}^{\infty }\sum_{m=1}^{n}mC_{n}^{m}B_{n}^{m,u} \end{array}$$

with

(28) $$\;C_{n}^{m}\;=\;\frac{2n+1}{n(n+1)}\frac{(n+|m|)!}{(n-|m|)!}$$

(29) $$A_{n}^{m,u}\;=\;A_{n}\left(g_{n,TM}^{m-1,u}g_{n,TM}^{m,u*}-g_{n,TM}^{-m,u}g_{n,TM}^{-m+1,u*}\right)\;+\;B_{n}\left(g_{n,TE}^{m-1,u}g_{n,TE}^{m,u*}-g_{n,TE}^{-m,u}g_{n,TE}^{-m+1,u*}\right)$$

(30) $$B_{n}^{m,u}\;=\;A_{n}\left({\left|g_{n,TM}^{m,u}\right|}^{2}-{\left|g_{n,TM}^{-m,u}\right|}^{2}\right)\;+\;B_{n}\left({\left|g_{n,TE}^{m,u}\right|}^{2}-{\left|g_{n,TE}^{-m,u}\right|}^{2}\right)$$

(31) $$\begin{array}{ccc} A_{n} & = & ℜ\left(a_{n}\right)-{\left|a_{n}\right|}^{2} \end{array}$$

(32) $$\begin{array}{ccc} B_{n} & = & ℜ\left(b_{n}\right)-{\left|b_{n}\right|}^{2}. \end{array}$$

## 3. Numerical Results and Discussion

The theory developed in the previous section was used to calculate the optical force and torque exerted on a graphene-coated gold nanosphere placed in a vector Bessel beam. The axial components of the optical force $F_{z}$ and torque $T_{z}$ are discussed, with emphasis on the effects of the beam order *l*, polarization, half-cone angle $\alpha_{0}$, and layer number *N*. In our calculation, the thickness of monolayer graphene was 0.335 nm and the layer number was *N*, so the thickness of the whole coating was $t_{G}=0.335\times N$ nm. The radius of the core was 10 nm. The beam center was assumed to be $\left(x_{0},y_{0},z_{0}\right)=\left(0,0,0\right)$, that is, the on-axis case was considered. Note that the transverse components ($F_{x}$, $F_{y}$, $T_{x}$ and $T_{y}$) and the off-axis case $\left(\left(x_{0},y_{0},z_{0}\right)\neq \left(0,0,0\right)\right)$ were also considered but they are not given in this paper.

### 3.1. Optical Force

**Figure 2:**
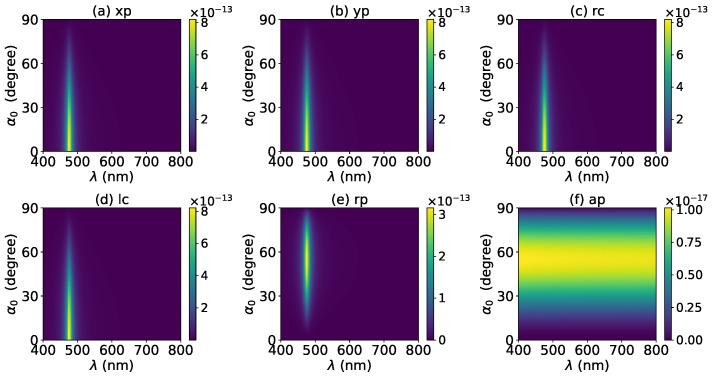
The axial optical force $F_{z}$ of a zeroth-order Bessel beam centered on a graphene-coated gold nanosphere. The layer number is N=0, which means the particle is a gold nanosphere without graphene coating. Panels (**a**–**f**) correspond to linear, circular, radial, and azimuthal polarizations, respectively. The titles of panels xp, yp, rc, lc, rp, and ap denote x, y, right circular, left circular, radial, and azimuthal polarizations, respectively.

The axial optical force exerted on a graphene-coated gold nanosphere was first investigated. Figure 2 shows the axial optical force $F_{z}$ on a graphene-coated gold nanosphere with layer number N=0 by a zeroth-order Bessel beam (l=0). Thus, the particle is a gold nanosphere without graphene coating. Figure 2a–f correspond to linear, circular, radial, and azimuthal polarizations, respectively. It can be seen that the axial optical force has an island formed by broad band peaks caused by a localized surface plasmon resonance (LSPR), which occurs due to the collective oscillations of free electrons when the particle is placed in the oscillating electric field of the incident beam. The islands for linear and circular polarizations (Figure 2a–d) are the same. The island for a radial polarization (Figure 2e) is located at the same wavelength as that for the linear and circular polarizations, since the resonance frequency should be independent of the polarization. However, the LSPR peak moves toward a larger half-cone angle $\alpha_{0}$. This can be explained from the dominant electric fields for various polarizations. As shown in Appendix A, the electric fields for linear, circular, and radial polarizations are dominated by the component including the term $J_{l}\left(\sigma \right)$. However, the dominant component for the radial polarization includes another term $P_{∥}$, which increases with $\alpha_{0}$. The same collective oscillations of free electrons need the same dominant oscillating electric field of the incident beams, so a larger $\alpha_{0}$ is necessary for the radial polarization. For the azimuthal polarization, the island disappears. This is because the weaker electric field, which is dominated by the term $J_{l\pm 1}\left(\sigma \right)$, leads to a weaker interaction between the free electrons and the incident beam.

**Figure 3:**
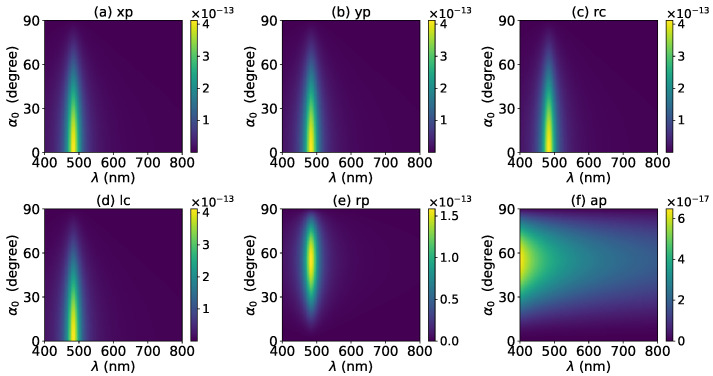
The same as in Figure 2, but with N=1.

**Figure 4:**
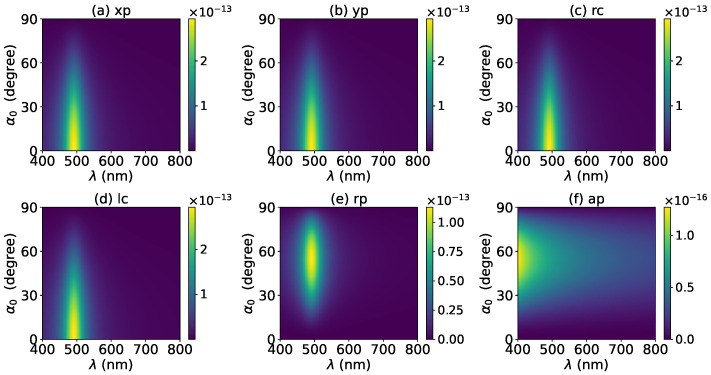
The same as in Figure 2, but with N=2.

**Figure 5:**
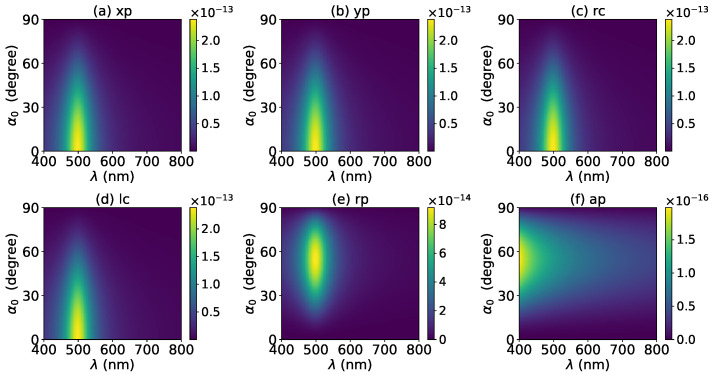
The same as in Figure 2, but with N=3.

**Figure 6:**
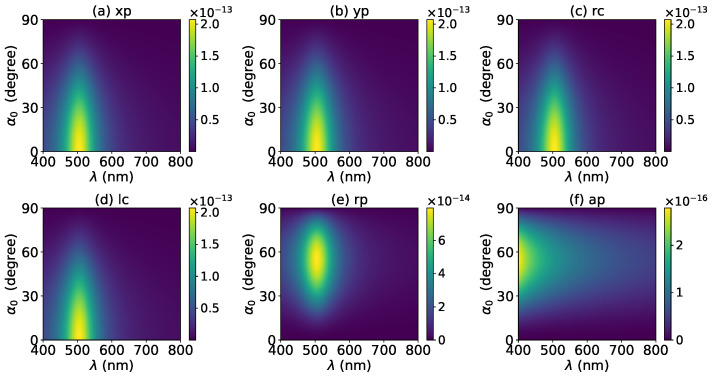
The same as in Figure 2, but with N=4.

The effects of increasing the layer number of graphene coating to N=1 on the axial optical force were investigated and are shown in Figure 3. A panel-to-panel comparison of Figure 2 and Figure 3 shows that with the increase of *N*, the LSPR peaks shift to the longer wavelength. This is mainly caused by the phase retardation effect with the increase of the graphene coating thickness, which is the same as the redshift of extinction spectra [57]. Therefore, we can use a laser beam with a longer wavelength to trap particles by increasing the graphene layers. Furthermore, since the increase of the coating thickness leads to the decrease of free electrons, which participate in the LSPR oscillation, the magnitude of the axial optical force becomes smaller. Meanwhile, the peaks are broader along both wavelength and half-cone angle directions, so we can trap the particle using a Bessel beam in a larger spectrum and half-cone angle range. As shown in Figure 4, Figure 5 and Figure 6, if the thickness of the graphene coating (layer number *N*) is further increased, the peaks shift to the longer wavelength, and the islands become wider.

**Figure 7:**
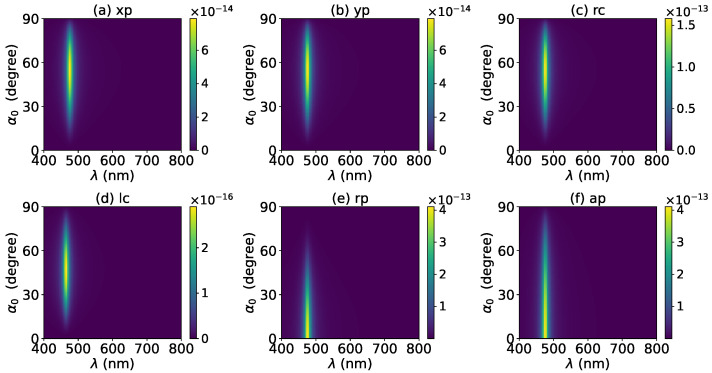
The axial optical force $F_{z}$ of a first-order Bessel beam centered on a graphene-coated gold nanosphere. The layer number is N=0, which means the particle is a gold nanosphere without graphene coating. Panels (**a**–**f**) correspond to linear, circular, radial, and azimuthal polarizations, respectively.

Next, the axial optical force exerted on a graphene-coated gold nanosphere by a first-order (l=1) vector Bessel beam was considered, with a particular emphasis on the effect of the graphene coating thickness. Figure 7 shows the axial optical force on a gold nanoparticle, namely, the layer number of graphene coating is N=0, by a first-order Bessel beam. Figure 7a–f correspond to linear, circular, radial, and azimuthal polarizations, respectively. A panel-to-panel comparison of Figure 2 and Figure 7 shows that the LSPR peaks for l=1 are located at the same wavelength as that for l=0, since the LSPR frequency is the intrinsic frequency of the particle and is independent of the incident beam. Comparing to the case of l=0, the LSPR peaks shift to a larger half-cone angle $\alpha_{0}$ for linear and circular polarizations, while the peaks shift to a smaller $\alpha_{0}$ for the radial polarization. This is because for l=1, the electric fields are dominated by the component including the term $J_{l-1}\left(\sigma \right)$. However, for linear and circular polarizations, a term $P_{∥}$ is included. Thus, a larger $\alpha_{0}$ is necessary for l=1 to generate the same dominant oscillating electric field of the incident beams as that for l=0, and to generate the same LSPR. For the radial polarization, in addition to the term $P_{∥}$, an additional term $\cot(\alpha_{0})$, which decreases with the increase of $\alpha_{0}$, is included. Thus, a smaller $\alpha_{0}$ is necessary. Note that the LSPR peaks for the azimuthal polarization can be seen. This is because, for l=1, the Bessel beam with the azimuthal polarization has a dominant electric field component including the term $J_{l-1}\left(\sigma \right)$. Since this electric field component has a term $\frac{P_{∥}}{\sin\alpha_{0}}=\frac{1}{1+\cos\alpha_{0}}$, which decreases with the increase of $\alpha_{0}$, the LSPR peaks are located at a smaller $\alpha_{0}$.

**Figure 8:**
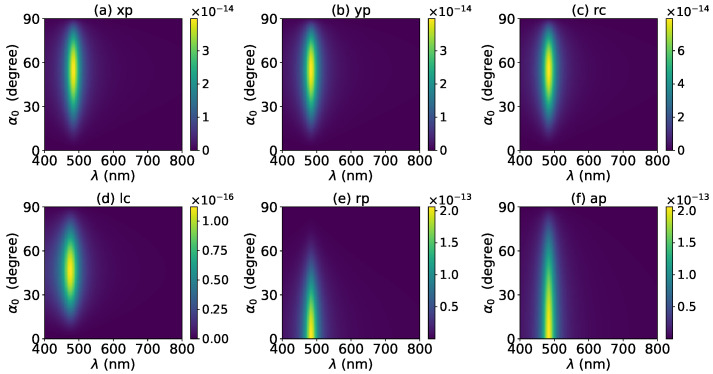
The same as in Figure 7, but with N=1.

**Figure 9:**
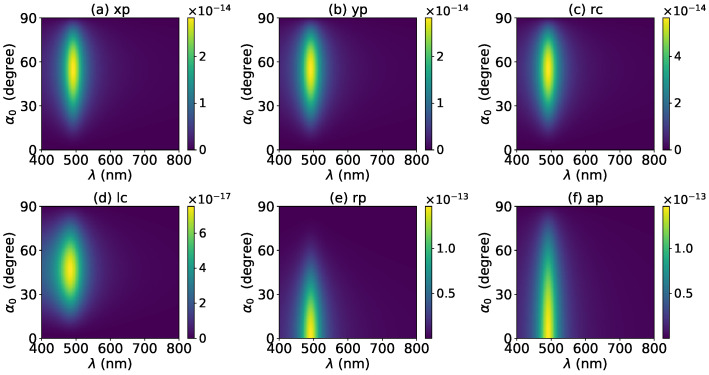
The same as in Figure 7, but with N=2.

**Figure 10:**
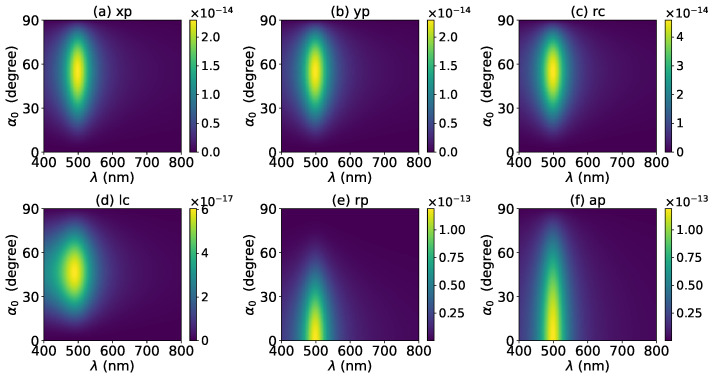
The same as in Figure 7, but with N=3.

**Figure 11:**
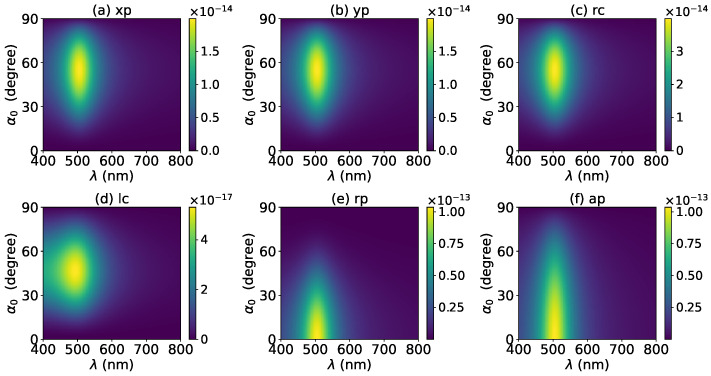
The same as in Figure 7, but with N=4.

The effects of increasing the graphene coating thickness (layer number *N*) on the axial optical force by a first-order vector Bessel beam were also investigated. Figure 8, Figure 9, Figure 10 and Figure 11 shows the axial optical force for layer number N=1∼4, respectively. From a panel-to-panel comparison of Figure 7, Figure 8, Figure 9, Figure 10 and Figure 11, it can be seen that with the increase of the graphene coating thickness (*N*), the LSPR peaks shift toward a longer wavelength and also become wider. Furthermore, with the increase of the *N*, the LSPR peaks cover a larger range of the half-cone angle, and the magnitude of the axial optical forces decrease. These characteristics can be explained by the same method used in the case of l=0.

**Figure 12:**
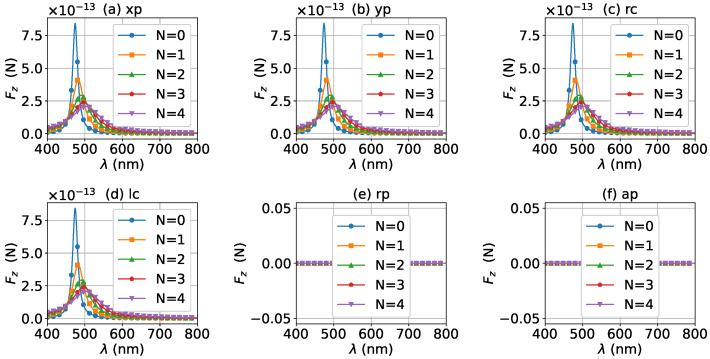
The axial optical force $F_{z}$ exerted on a graphene-coated gold nanosphere by a zeroth-order vector Bessel beam as a function of wavelength with various layer numbers *N*. The half-cone angle is $\alpha_{0}=0^{\circ }$.

**Figure 13:**
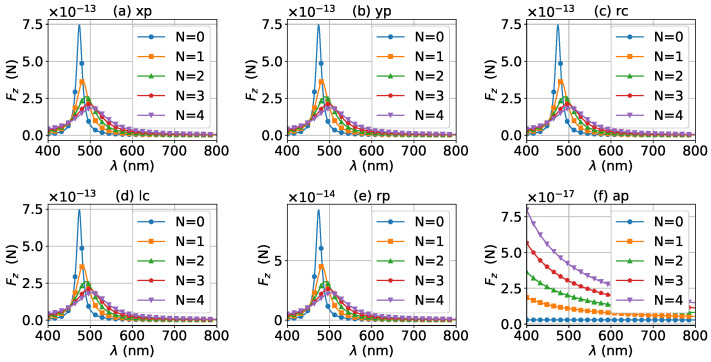
The same as in Figure 12, but with $\alpha_{0}=20^{\circ }$.

**Figure 14:**
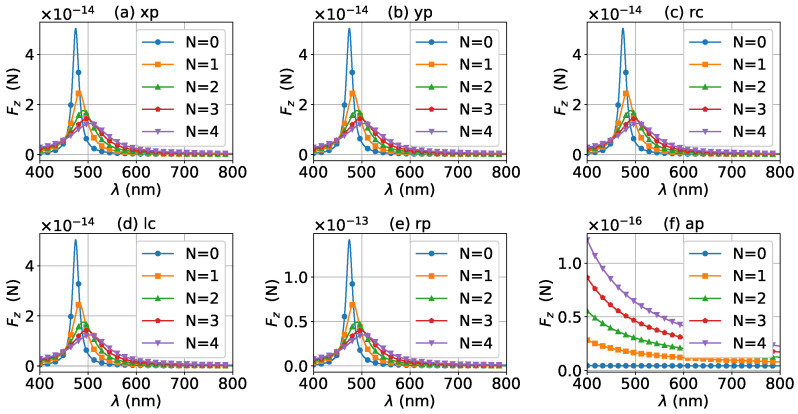
The same as in Figure 12, but with $\alpha_{0}=80^{\circ }$.

For a better quantitative understanding of the effects of graphene coating thickness, we calculated the axial optical force spectrum, namely the axial optical force $F_{z}$ versus the wavelength λ, for a single half-cone angle $\alpha_{0}$ with layer number *N* being a parameter. Figure 12, Figure 13 and Figure 14 display the results for $\alpha_{0}=0^{\circ }$, $20^{\circ }$, and $80^{\circ }$, respectively. Note that in our calculation, only the on-axis ($x_{0}=y_{0}=z_{0}=0$) was considered. Since zeroth-order Bessel beams with radial and azimuthal polarizations have a zero central electric field, the optical force vanishes as shown in Figure 12e,f. The Bessel beams with linear and circular polarizations have a similar optical force spectrum. For N=0, the optical force spectrum has an LSPR peak at about λ=474 nm. If the layer number increases to N=1, the peak shifts to about λ=482 nm. If the thickness increases further, the peaks for N=2, 3, and 4 are at λ=490 nm, 497 nm, and 503 nm, respectively. In the meanwhile, the magnitudes of $F_{z}$ decrease with the increasing of *N*, and the LSPR peaks become wider once the graphene coating thickness is added. As shown in Figure 13 and Figure 14, the LSPR peaks for $\alpha_{0}=20^{\circ }$ and $80^{\circ }$ are located at the same wavelength as that for $\alpha_{0}=0^{\circ }$ as shown in Figure 12. This means that the increasing of the half-cone angle does not affect the LSPR frequency (or wavelength). For the radial and azimuthal polarizations, a zeroth-order Bessel beam with $\alpha_{0}\neq 0^{\circ }$ has a nonzero central field, so the axial optical forces are not zero. Meanwhile, since the electric field for azimuthal polarization is weak, the optical forces are smaller than that for other polarizations, and the LSPR vanishes. Note that the increase of the half-cone angle affects the magnitude of the axial optical force.

**Figure 15:**
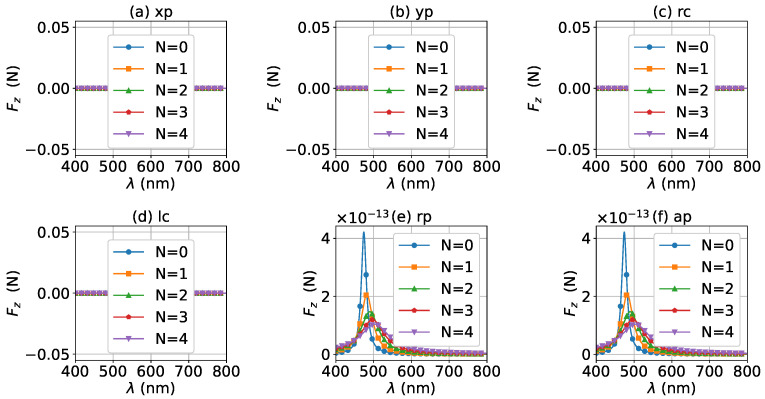
The axial optical force $F_{z}$ exerted on a graphene-coated gold nanosphere by a first-order vector Bessel beam as a function of wavelength with various layer numbers *N*. The half-cone angle is $\alpha_{0}=0^{\circ }$.

**Figure 16:**
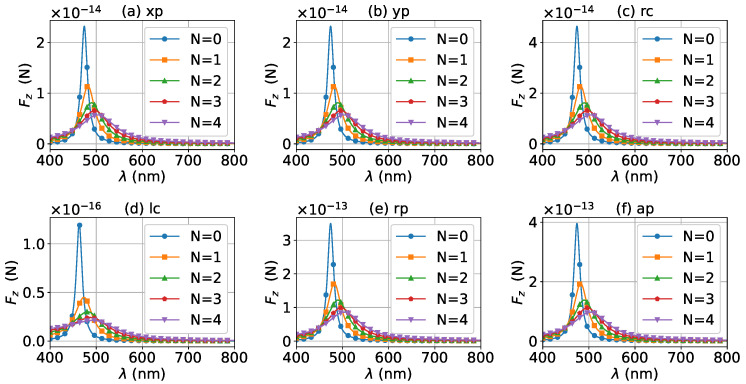
The same as in Figure 15, but with $\alpha_{0}=20^{\circ }$.

**Figure 17:**
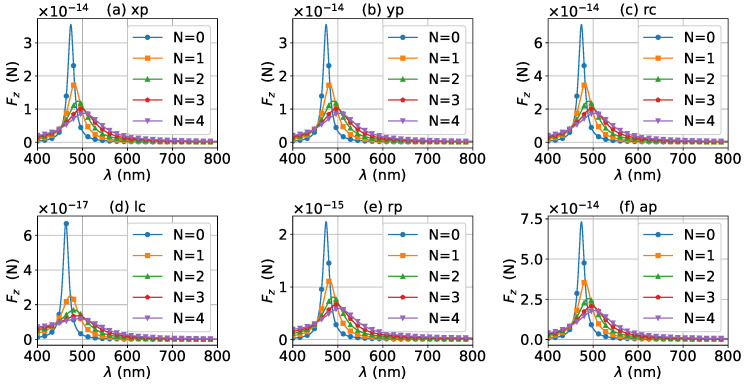
The same as in Figure 15, but with $\alpha_{0}=80^{\circ }$.

The axial optical force spectrum for a first-order Bessel beam was also investigated, and the results for $\alpha_{0}=0^{\circ }$, $20^{\circ }$, and $80^{\circ }$ are given in Figure 15, Figure 16 and Figure 17, respectively. In general, the positions of the LSPR peaks are the same as those for zeroth-order Bessel beams, since the LSPR is independent of the incident beam. Since first-order Bessel beams with $\alpha_{0}=0^{\circ }$ and linear and circular polarizations have null central fields, the axial optical forces are zero as shown in Figure 15a–d. On the contrary, the axial optical forces for the radial and azimuthal polarizations are not zero, since the corresponding central fields are not zero. It can be seen from Figure 16 and Figure 17 that the LSPR peaks can be observed for all $\alpha_{0}\neq 0^{\circ }$ and polarizations. With the increase of the thickness of the graphene coating, the LSPR peaks shift to a longer wavelength, and the axial optical force becomes weaker. Note that the half-cone angle $\alpha_{0}$ affects the magnitude of the axial optical force.

**Figure 18:**
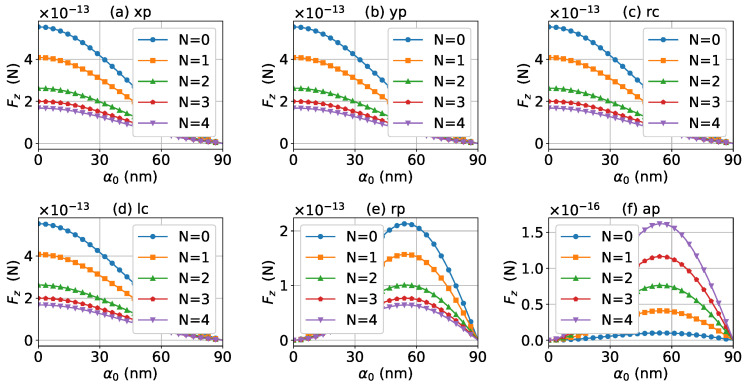
The axial optical force $F_{z}$ exerted on a graphene-coated gold nanosphere by a zeroth-order vector Bessel beam as a function of half-cone angle $\alpha_{0}$ with various layer numbers *N*. The wavelength is λ=480 nm.

**Figure 19:**
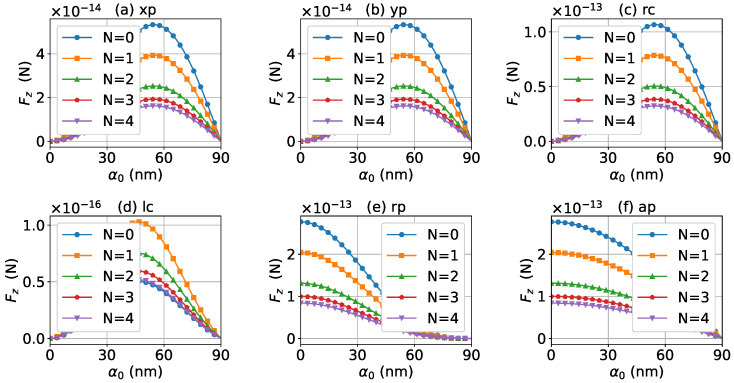
The same as in Figure 18, but with l=1.

As discussed above, the half-cone angle $\alpha_{0}$ can affect the magnitude of the axial optical force. For a better quantitative understanding, we calculated the axial optical force versus the half-cone angle $\alpha_{0}$. Figure 18 and Figure 19 display the results for zeroth- and first-order Bessel beams, respectively. In the calculation, the wavelength of the incident beam was λ=480 nm, which is close to the LSPR peak for a gold nanosphere without graphene coating. As shown in Figure 18, the optical force decreases with the increase of $\alpha_{0}$, if the incident beams are zeroth-order Bessel beams with linear and circular polarizations, while for radial and azimuthal polarizations, the axial optical forces increase first, reaches the maximum at about $\alpha_{0}=55^{\circ }$, and then decreases. The thickness of the graphene coating decreases the number of free electrons participating in the LSPR oscillation, and eventually makes the optical force become smaller. If the incident beam is a first-order Bessel beam with linear and circular polarizations, the optical force increases first, reaches its maximum at about $\alpha_{0}=55^{\circ }$, and then decreases. For the radial and azimuthal polarizations, the optical forces decrease with the increase of $\alpha_{0}$. Similar to the case of a zeroth-order Bessel beam, with the increase of the thickness of the graphene coating, the optical force becomes smaller.

### 3.2. Optical Torque

The optical torque on a graphene-coated gold nanosphere by a vector Bessel beam was investigated. Though we calculated all three components, $T_{x}$, $T_{y}$, and $T_{z}$, of the optical torque, we only discuss the axial component $T_{z}$ in this paper, since we mainly focus on the effects of graphene coating thickness and beam parameters including half-cone angle, order, and polarization on optical torque. The radius of the core was 10 nm.

**Figure 20:**
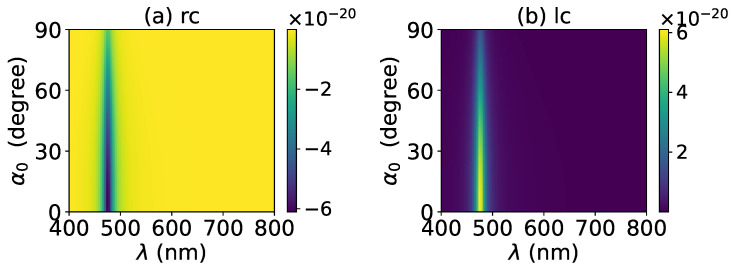
The axial optical torque $T_{z}$ of a zeroth-order Bessel beam centered on a graphene-coated gold nanosphere. The layer number is N=0, which means the particle is a gold nanosphere without graphene coating. Panels (**a**–**f**) correspond to linear, circular, radial, and azimuthal polarizations, respectively.

**Figure 21:**
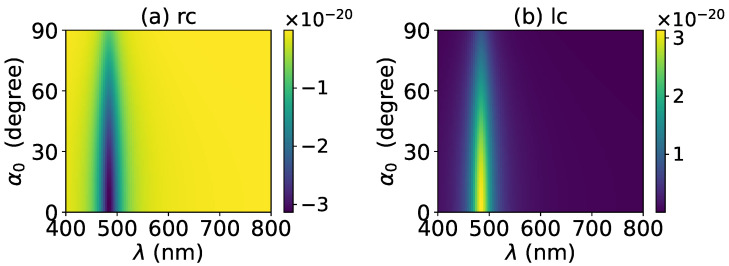
The same as in Figure 20, but with N=1.

**Figure 22:**
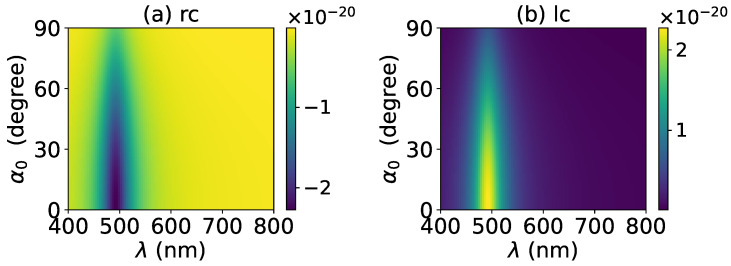
The same as in Figure 20, but with N=2.

**Figure 23:**
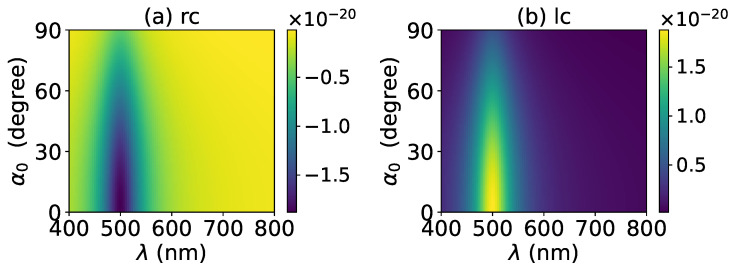
The same as in Figure 20, but with N=3.

**Figure 24:**
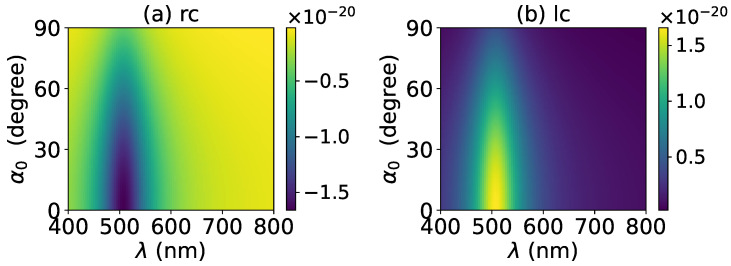
The same as in Figure 20, but with N=4.

The axial optical torque by a zeroth-order Bessel beam was first calculated. Figure 20, Figure 21, Figure 22, Figure 23 and Figure 24 display the results for a graphene-coated gold nanosphere with layer numbers N=0∼4, respectively. Since zeroth-order Bessel beams with linear, radial, and azimuthal polarizations carry no angular momentum, the particle does not experience any optical torque. Since a Bessel beam with circular polarization carries a spin angular momentum, it exerts an axial optical torque on the particle and makes the particle rotate around its center of mass. Therefore, only the axial optical torques for circular polarizations are given in Figure 20, Figure 21, Figure 22, Figure 23 and Figure 24. Panels (a) and (b) of each figure correspond to the right and left circular polarizations, respectively. In general, we have $T_{z}^{rc}=-T_{z}^{lc}$. This means that the axial optical torques for the left and right polarizations have the same magnitudes but opposite directions. Similar to the optical force, we can see the LSPR peaks of the optical torque. With the increasing of the graphene coating thickness (layer number *N*), the peaks shift to a longer wavelength. For N=0∼4, the LSPR wavelengths are about λ=474 nm, 482 nm, 490 nm, 497 nm, and 503 nm, respectively. A comparison shows that these LSPR wavelengths are the same as those for optical forces. The reason is that both optical force and torque are based on the same particle scattering problem, and the LSPR wavelength is determined by the scattering of particles. Furthermore, with the increase of the graphene coating thickness, the LSPR peak becomes wider, and the axial optical torque becomes smaller.

**Figure 25:**
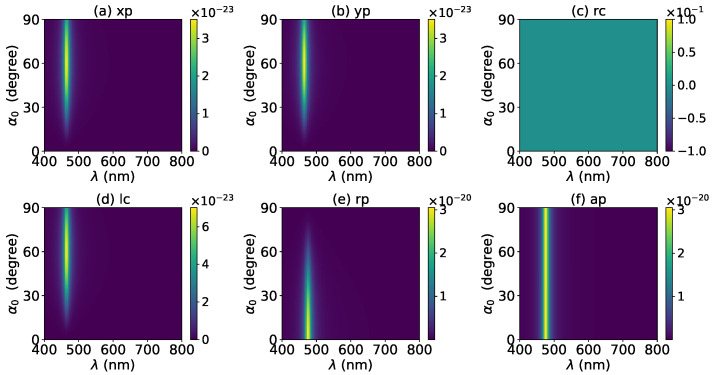
The axial optical torque $T_{z}$ of a first-order Bessel beam centered on a graphene-coated gold nanosphere. The layer number is N=0, which means the particle is a gold nanosphere without graphene coating. Panels (**a**–**f**) correspond to linear, circular, radial, and azimuthal polarizations, respectively.

**Figure 26:**
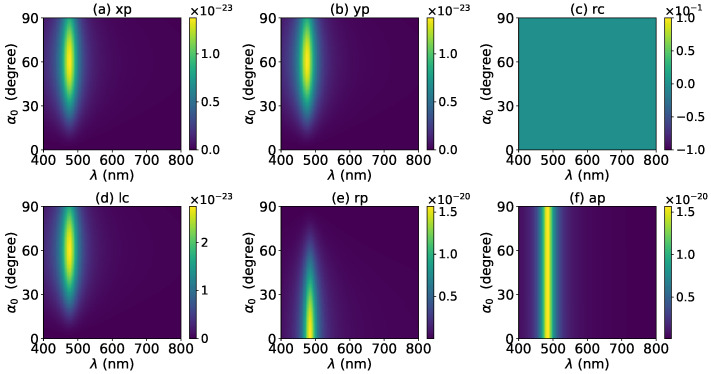
The same as in Figure 25, but with N=1.

**Figure 27:**
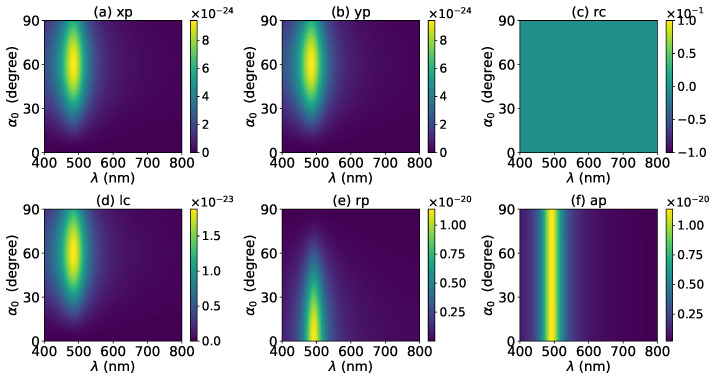
The same as in Figure 25, but with N=2.

**Figure 28:**
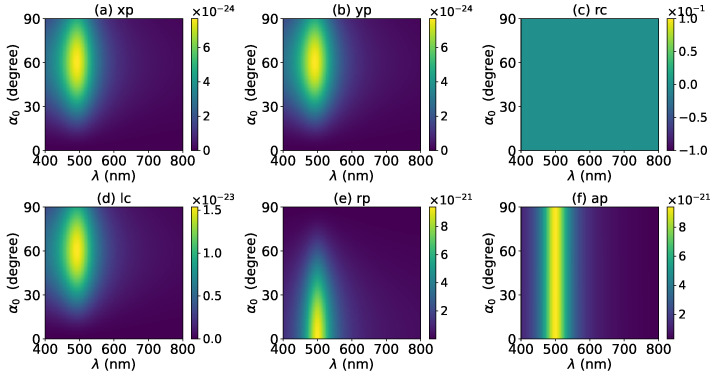
The same as in Figure 25, but with N=3.

**Figure 29:**
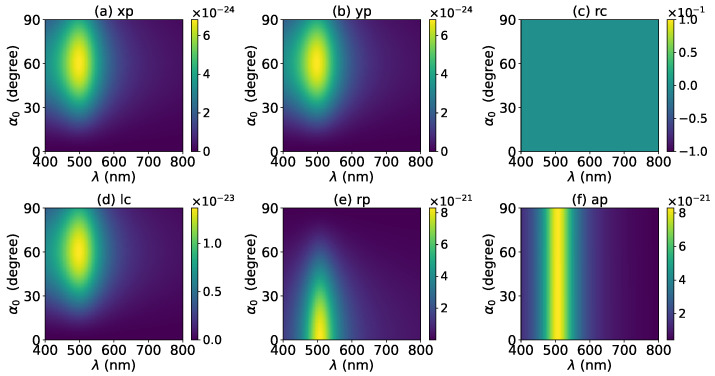
The same as in Figure 25, but with N=4.

The effect of increasing the beam order to l=1 on the axial optical torque was investigated and is displayed in Figure 25, Figure 26, Figure 27, Figure 28 and Figure 29. Since a first-order Bessel beam carries an orbit angular momentum, the axial optical torques generated by Bessel beams with linear, radial, and azimuthal polarizations are nonzero, as shown in panels (a), (b), (e) and (d) of Figure 25, Figure 26, Figure 27, Figure 28 and Figure 29. Bessel beams with a circular polarization also carry a spin angular momentum. Since each photon of a first-order Bessel beam with a circular polarization carries an orbit angular momentum *ℏ* and a spin angular momentum ±ℏ (+for left circular polarization, and − for right circular polarization), the total angular momentum carried by each photon is 2ℏ (left circular polarization) or 0 (right circular polarization). Therefore, the axial optical torque for the right circular polarization vanishes as shown in panel (c) of Figure 25, Figure 26, Figure 27, Figure 28 and Figure 29, while the optical torque for the left circular polarization is twice as great as that for the linear polarization. As the graphene coating thickness increases, the LSPR peaks become wider, and the axial optical torque becomes smaller. It is very interesting that the increase of the graphene coating thickness does not shift the LSPR wavelength.

## 4. Conclusions

The optical force and torque on a graphene-coated gold nanosphere by a vector Bessel beam were investigated in the framework of the generalized Lorenz–Mie theory. The dielectric function of the gold core was described by the Drude–Sommerfeld mode, and that of the graphene coating was given by the Lorentz–Drude model. The coating was a *N*-layered graphene. The axial optical force and torque were numerically discussed, with particular emphasis on the effects of the graphene coating thickness (layer number *N*) and beam parameters, including the half-cone angle $\alpha_{0}$, order *l*, and polarizations. Numerical results showed that LSPR peaks can be seen when a graphene-coated gold nanosphere is placed in a vector Bessel beam. With the increase of the graphene coating thickness, the LSPR peaks shifted toward a longer wavelength, and they became wider. Furthermore, the increase of the graphene coating thickness made the optical force and torque become smaller. Furthermore, the LSPR peaks were very sensitive to the beam parameters $\alpha_{0}$, order *l*, and polarizations. Thus, by choosing suitable beam parameters and graphene coating thickness, we can provide a better manipulation and rotation of the particle at desirable wavelengths.

## Data Availability

Not applicable.

## Appendix A. Electric Fields of Vector Bessel Beams with Various Polarizations

- x−polarization
  (A1) $$\left\{\begin{array}{c} E_{x}^{x}\;=\;E_{0}e^{ik_{z}z}e^{il\phi }i^{l}\left[J_{l}+\frac{1}{2}\left(J_{l+2}e^{2i\phi }+J_{l-2}e^{-2i\phi }\right)P_{⊥}\right]\\ E_{y}^{x}\;=\;E_{0}e^{ik_{z}z}e^{il\phi }i^{l-1}\left(\frac{1}{2}\right)\left(J_{l+2}e^{2i\phi }-J_{l-2}e^{-2i\phi }\right)P_{⊥}\\ E_{z}^{x}\;=\;E_{0}e^{ik_{z}z}e^{il\phi }i^{l-1}\left(J_{l+1}e^{i\phi }-J_{l-1}e^{-i\phi }\right)P_{∥} \end{array}\right.$$
  where $E_{0}=\pi E_{pw_{0}}\left(1+\cos\alpha_{0}\right)$ with $E_{pw_{0}}$ the amplitude of the plane wave forming the Bessel beam. $J_{l}\left(\;\cdot \;\right)$ is the $l^{th}$-order Bessel function, and its argument $\sigma =k_{\rho }\rho =k\rho \sin\alpha_{0}$ has been omitted. $P_{⊥}=\left(\frac{1-\cos\alpha_{0}}{1+\cos\alpha_{0}}\right)$ and $P_{∥}=\left(\frac{\sin\alpha_{0}}{1+\cos\alpha_{0}}\right)$.
- y−polarization
  (A2) $$\left\{\begin{array}{c} E_{x}^{y}\;=\;E_{0}e^{ik_{z}z}e^{il\phi }i^{l-1}\left(\frac{1}{2}\right)\left(J_{l+2}e^{2i\phi }-J_{l-2}e^{-2i\phi }\right)P_{⊥}\\ E_{y}^{y}\;=\;E_{0}e^{ik_{z}z}e^{il\phi }i^{l}\left[J_{l}-\frac{1}{2}\left(J_{l+2}e^{2i\phi }+J_{l-2}e^{-2i\phi }\right)P_{⊥}\right]\\ E_{z}^{y}\;=\;-E_{0}e^{ik_{z}z}e^{il\phi }i^{l}\left(J_{l+1}e^{i\phi }+J_{l-1}e^{-i\phi }\right)P_{∥} \end{array}\right.$$
- Circular polarization
  (A3) $$\left\{\begin{array}{c} E_{x}^{circ}\;=\;\left(E_{x}^{x}\pm iE_{x}^{y}\right)/\sqrt{2}\\ E_{y}^{circ}\;=\;\left(E_{y}^{x}\pm iE_{y}^{y}\right)/\sqrt{2}\\ E_{z}^{circ}\;=\;\left(E_{z}^{x}\pm iE_{z}^{y}\right)/\sqrt{2} \end{array}\right.$$
  where + and − correspond to left and right polarizations, respectively.
- Radial polarization
  (A4) $$\left\{\begin{array}{c} E_{x}^{rp}\;=\;-E_{0}e^{ik_{z}z}e^{il\phi }i^{l-1}\left(J_{l+1}e^{i\phi }-J_{l-1}e^{-i\phi }\right)P_{∥}\cot\alpha_{0}\\ E_{y}^{rp}\;=\;E_{0}^{ik_{z}z}e^{il\phi }i^{l}\left(J_{l+1}e^{i\phi }+J_{l-1}e^{-i\phi }\right)P_{∥}\cot\alpha_{0}\\ E_{z}^{rp}\;=\;-2P_{∥}E_{0}e^{ik_{z}z}e^{il\phi }i^{l}J_{l} \end{array}\right.$$
- Azimuthal polarization
  (A5) $$\left\{\begin{array}{c} E_{x}^{ap}\;=\;-E_{0}^{ik_{z}z}e^{il\phi }i^{l}\left(J_{l+1}e^{i\phi }+J_{l-1}e^{-i\phi }\right)P_{∥}/\sin\alpha_{0}\\ E_{y}^{ap}\;=\;-E_{0}e^{ik_{z}z}e^{il\phi }i^{l-1}\left(J_{l+1}e^{i\phi }-J_{l-1}e^{-i\phi }\right)P_{∥}/\sin\alpha_{0}\\ E_{z}^{ap}\;=\;0 \end{array}\right.$$

## Appendix B. BSCs for Vector Bessel Beam with Various Polarizations

(A6) $$g_{n,TM}^{m,u}\;=\;\left\{\begin{array}{cc} \frac{n(n+1)}{i^{n+1}\left(2n+1\right)}q_{mn}^{u} & m\geq 0\\ \frac{1}{i^{n+1}{\left(-1\right)}^{m}}\frac{(n+m)!}{(n-m)!}\frac{n(n+1)}{\left(2n+1\right)}q_{mn}^{u} & m<0 \end{array}\right.$$

(A7) $$g_{n,TE}^{m,u}\;=\;\left\{\begin{array}{cc} \frac{-in(n+1)}{i^{n+1}\left(2n+1\right)}p_{mn}^{u} & m\geq 0\\ \frac{-i}{i^{n+1}{\left(-1\right)}^{m}}\frac{(n+m)!}{(n-m)!}\frac{n(n+1)}{\left(2n+1\right)}p_{mn}^{u} & m<0 \end{array}\right.$$

with

(A8) $$p_{mn}^{u}\;=\;-4i^{n+1}D_{mn}e^{-ik_{z}z_{0}}\left[\pi_{mn}\left(\cos\alpha_{0}\right)I_{+}^{u}+\tau_{mn}\left(\cos\alpha_{0}\right)I_{-}^{u}\right]$$

(A9) $$q_{mn}^{u}\;=\;-4i^{n+1}D_{mn}e^{-ik_{z}z_{0}}\left[\tau_{mn}\left(\cos\alpha_{0}\right)I_{+}^{u}+\pi_{mn}\left(\cos\alpha_{0}\right)I_{-}^{u}\right]$$

- x−polarization
  (A10) $$I_{\pm }^{xp}\;=\;\pi \left[i^{1+l-m}e^{i\left(1+l-m\right)\phi_{0}}J_{1+l-m}\left(\sigma_{0}\right)\pm i^{-1+l-m}e^{i\left(-1+l-m\right)\phi_{0}}J_{-1+l-m}\left(\sigma_{0}\right)\right]$$
  where, $\phi_{0}=\arctan\left(y_{0}/x_{0}\right)$, and $\sigma_{0}=-k\rho_{0}\sin\alpha_{0}$.
- y−polarization
  (A11) $$I_{\pm }^{yp}\;=\;-i\pi \left[i^{1+l-m}e^{i\left(1+l-m\right)\phi_{0}}J_{1+l-m}\left(\sigma_{0}\right)\mp i^{-1+l-m}e^{i\left(-1+l-m\right)\phi_{0}}J_{-1+l-m}\left(\sigma_{0}\right)\right]$$
- Circular polarization
  (A12) $$\left\{\begin{array}{c} p_{mn}^{cp}\\ q_{mn}^{cp} \end{array}\right\}\;=\;\left\{\begin{array}{c} p_{mn}^{xp}\\ q_{mn}^{xp} \end{array}\right\}\;\pm \;i\left\{\begin{array}{c} p_{mn}^{yp}\\ q_{mn}^{yp} \end{array}\right\}$$
- Radial polarization
  (A13) $$\begin{array}{c} I_{+}^{rp}\;=\;2\pi i^{l-m}e^{i\left(l-m\right)\phi_{0}}J_{l-m}\left(\sigma_{0}\right)\\ I_{-}^{rp}\;=\;0 \end{array}$$
- Azimuthal polarization
  (A14) $$\begin{array}{c} I_{+}^{ap}\;=\;0\\ I_{-}^{ap}\;=\;-i2\pi i^{l-m}e^{i\left(l-m\right)\phi_{0}}J_{l-m}\left(\sigma_{0}\right) \end{array}$$
